# Editorial: Targeting signals in protein trafficking and transport

**DOI:** 10.3389/fphys.2023.1338852

**Published:** 2023-12-06

**Authors:** Markus Kunze, Nathan N. Alder, Inhwan Hwang, Guillaume Roussel, Andrey L. Karamyshev

**Affiliations:** ^1^ Department of Pathobiology of the Nervous System, Center for Brain Research, Medical University of Vienna, Vienna, Austria; ^2^ Department of Molecular and Cell Biology, University of Connecticut, Storrs, CT, United States; ^3^ Department of Life Sciences, Pohang University of Science and Technology, Pohang, Republic of Korea; ^4^ Laboratory of Molecular Bacteriology, Department of Microbiology, Immunology, and Transplantation, Rega Institute, Katholieke Universiteit-Leuven, Leuven, Belgium; ^5^ Department of Cell Biology and Biochemistry, Texas Tech University Health Sciences Center, Lubbock, TX, United States

**Keywords:** protein transport, targeting signals, mitochondria, chloroplasts, endoplasmic reticulum, peroxisomes, bacterial secretion

*“This Research Topic of Frontiers in Physiology is dedicated to the memory of Professor Anastassios (Tassos) Economou, one of the guest editors of this issue, who recently passed away during its formation*.”

## Introduction

The distribution of proteins among the different compartments of cells and the ability to export proteins to the extracellular space is critically dependent on protein transport processes. In most cases these processes are governed by the interaction between targeting signals encoded in the primary sequence of proteins to be transported (cargo proteins) and receptor proteins linking them with the general transport machinery. This Research Topic is a collection of original articles and reviews covering transport processes into peroxisomes, mitochondria and chloroplasts, into the endoplasmic reticulum (ER) and across the secretory pathway, and protein secretion in bacteria. Remarkably, some targeting signals, particularly the N-terminal ones, are structurally similar, which raises questions of the relation between similarity and specificity (Kunze and Berger, 2015) and the conservation through evolution.

### Peroxisomes

Peroxisomes are single membrane bound organelles with two types of peroxisomal targeting signals for matrix proteins (PTS1 and PTS2) and another for peroxisomal membrane proteins (mPTS), which are recognized by the receptor proteins PEX5, PEX7 together with a co-receptor, and PEX19, respectively (Rudowitz and Erdmann, 2023). Krishna et al. describe targeting signals encoded in the peroxisomal membrane protein PEX11 from the protozoon *Trypanosoma brucei*, which not only has binding sites for the cognate peroxisomal receptor PEX19 but also for the mitochondrial receptor TOM20 although mitochondrial targeting is only observed when no peroxisomes are present. The first comprehensive inventory of peroxisomal proteins of the zebrafish (*Danio rerio*) by Kamoshita et al. reveals extensive similarity to the mammalian peroxisomal proteome and of the PTS1 and PTS2 motifs encoded in the homologous enzymes, which confirms *D. rerio* as promising model organism for chordates. Based on a PTS2-tagged EGFP reporter protein Lu et al. studied the peroxisomal dynamics in the fungus *Alternaria alternata* as a developmentally interesting model system for rapidly changing organismal states confirming previous studies on the relevance of peroxisomes for fungal developmental and infectious processes.

### Mitochondria

Mitochondria contain two membranes and several distinct subcompartments, each one with its own defined set of proteins (Iovine et al., 2021). These organelles have their own genome and biosynthetic machinery to produce some resident proteins. But the vast majority of mitochondrial proteins are encoded in nuclear DNA, synthesized on cytosolic ribosomes, and trafficked to the organelle. Targeting mitochondrial proteins to their correct subcompartments requires a diversity of import pathways, each one having its own type of targeting signal. Most nuclear-encoded proteins, however, are directed to mitochondria by N-terminal presequences that engage the TOM and TIM23 complexes of the outer and inner mitochondrial membranes, respectively. In this Research Topic, Genge and Mokranjac review what is currently known about the import of presequence-containing proteins into mitochondria with particular emphasis on the cooperation of the TOM and TIM23 complexes. Reed et al. review evidence for a more non-canonical role of TIM23-mediated import in neurodegenerative diseases, describing how amyloidogenic proteins bearing cryptic targeting signals may import into mitochondria as part of the pathogenic process, or perhaps as a quality control mechanism.

### Chloroplasts

Chloroplasts are an endosymbiotic organelle found in plants and algae. For their function, chloroplasts contain a large number of proteins, with estimates ranging from 2,000 to 5,000 different proteins. Over 95% of these proteins are targeted from the cytosol to various suborganellar compartments after translation (Peltier et al., 2000; Schleiff and Becker, 2011). The protein targeting mechanisms for chloroplasts are complex and diverse; each of the suborganellar compartments of chloroplasts employs specific protein targeting mechanisms. In this Research Topic, Jeong et al. review the diverse nature of transit peptides for specific targeting to chloroplasts and also provide analysis on how the targeting specificity is determined between chloroplasts and mitochondria. Zheng et al. summarize the properties and significance of liquid-liquid phase separation in the sorting of chloroplast twin arginine transport substrate proteins. Zhu et al. review the various protein transport systems from the chloroplast stroma to the thylakoid membrane and also describe the targeting of chloroplast-encoded proteins to the thylakoid membranes. Finally, Ballabani et al. review the common features of targeting sequences in routing preproteins to and across the chloroplast envelope as well as the thylakoid membrane and lumen. They also summarize recent findings on components of the import machinery at outer and inner envelopes, thylakoid membranes, and stroma.

### Secretory pathway

In humans, about 30%–40% of proteins are secretory and membrane proteins. These proteins need to be transported to different cellular organelles, inserted into membranes or transported outside of the cells. Many of these proteins use signal recognition particle (SRP) and SEC61 complex in ER for their transport (Kellogg et al., 2021). This process is tightly regulated, and its dysregulation is associated with multiple diseases. Secretory proteins have N-terminal targeting signals called signal peptides which are recognized by SRP, and mutations in them often lead to diseases (Gutierrez Guarnizo et al., 2023). In this Research Topic, Lang et al. review molecular mechanisms of ER protein targeting and translocation and analyze signal peptide features that determine the specificity of protein transport. Karamysheva and Karamyshev describe how cells protect themselves from aberrant secretory proteins by activating the RAPP protein quality control on the ribosome and discuss molecular mechanisms of human diseases associated with dysregulation of protein transport. Štepihar et al. focus on the late steps in protein transport—cell-specific secretory granule sorting mechanism and role of MAGEL2 protein in regulated secretion.

### Bacterial transport

Amongst the numerous secretion systems in bacteria, each secretory protein follows a dedicated pathway and therefore need to indicate to the cell its journey and destination (Loos et al., 2019). Evolution selected the signal peptide, a short amino-acid sequence, to direct the client to the appropriate secretion system. The review from Kaushik et al. examines in detail the complete journey of the signal peptide during secretion; from the cytoplasm where it delays the folding, then to the network of chaperones sorting it to the appropriate secretion system to be exported or inserted in the membrane; and finally, its degradation by the signal peptidase; and how this critical step can be used as a novel strategy for antibiotics development. While the key role of the signal peptide has largely been attributed to its amino acid sequence, the work of Spitz et al. on signal peptides from clients of the Type 1 secretion system introduces an interesting concept where a structural feature, a conserved amphipathic helix, is encoded in the signal peptide and is more important than the sequence itself.

Altogether, this Research Topic allows an up-to-date synopsis of various transport systems covering all phylogenetic kingdoms and providing a plethora of information on different targeting signals, the cognate receptor proteins, and their relevance for complex transport processes. Highlighting the benefits of a systematic analysis of the vast majority of proteins harboring the same type of targeting signal either by experimental approaches or by computational prediction of organellar proteomes from different species and their comparison, fostering a comparison of targeting signals directing proteins to different compartments in spite of similar structural properties, and emphasizing the relevance of protein transport systems and their associated quality controls for human diseases, this Research Topic pinpoints critical research questions of the field of protein transport today.

## In memoriam

Professor Economou was a renowned scientist in bacterial protein secretion, known for its significant and numerous contributions to the Sec and Type III translocase mechanisms. Native from Alexandroupolis in Greece, Tassos graduated in Biology in Thessaloniki, and then earned his PhD in Molecular Microbiology in 1990 from the John Innes Institute and University of East Anglia in the United-Kingdom. He then pursued his career as a postdoctoral researcher at the University of California in Los Angeles (United States), and then the Medical School of Dartmouth (United States). In 1999, he established his lab at the Institute of Molecular Biology and Biotechnology—Foundation of Research and Technology (IMBB-FORTH) at the University of Crete in Greece, where he also founded the MINOTECH Biotechnology company and Proteomics facility, and acted as a consultant for Pfizer and Integrated Genomics. Tassos played a significant role in the development and recognition of IMBB-FORTH as a center of excellence and led to extensive funding for large European and international programs in Greece. In 2013, Tassos changed to a more temperate climate and moved his lab to Belgium. He became professor of Molecular Biology and Biochemistry and the Head of the Molecular Bacteriology division, at the REGA institute, KU Leuven—Since his first publication in 1986, he collaborated on over 130 research articles, making notable contributions to top-ranking scientific journals such as Cell, Nature, and Science. He had an extraordinarily wide knowledge of the field of molecular biology, specifically on the molecular basis of protein secretion and folding. Over the years he created a unique infrastructure that encompasses not only cutting-edge molecular biology but also a multi-disciplinary biophysical toolset including single-molecule FRET and hydrogen deuterium exchange mass spectrometry, using his words, “A unique combination of tools that allow us to dissect the intricate mechanisms involved in protein secretion”. Tassos’ deep involvement in science extends beyond his publications, involving active participation in departmental boards, advisory committees, and diligent service as a reviewer and editor for renowned scientific journals. He had an extraordinary passion for science which was a profound source of inspiration and motivation for his students and collaborators. He mentored his students with the utmost dedication and fervor, instilling them with a lifelong love for science and the pursuit of knowledge. Outside of his research lab, Tassos was a great father of three kids, a dedicated husband for over three decades, a caring friend, and an excellent cook (always bringing Greek delicacies during the lab dinners). His legacy as a mentor and scientist will continue to inspire the next generation of scientists, forever grateful for the passion and enthusiasm with which he guided them on their scientific and life journeys.
